# Validation of a Three-Dimensional Weight-Bearing Measurement Protocol for Medial Open-Wedge High Tibial Osteotomy

**DOI:** 10.3390/jcm13051280

**Published:** 2024-02-23

**Authors:** Sandro Hodel, Julian Hasler, Tabitha Arn Roth, Andreas Flury, Cyrill Sutter, Sandro F. Fucentese, Philipp Fürnstahl, Lazaros Vlachopoulos

**Affiliations:** 1 Department of Orthopedics, Balgrist University Hospital, Forchstrasse 320, 8008 Zurich, Switzerlandandreas.flury@balgrist.ch (A.F.);; 2 Research in Orthopedic Computer Science, Balgrist University Hospital, Forchstrasse 320, 8008 Zurich, Switzerlandphilipp.fuernstahl@balgrist.ch (P.F.)

**Keywords:** leg alignment, automatization, three-dimensional, HTO, MOWHTO, planning

## Abstract

Three-dimensional (3D) deformity assessment and leg realignment planning is emerging. The aim of this study was to (1) validate a novel 3D planning modality that incorporates the weight-bearing (WB) state (3D WB) by comparing it to existing modalities (3D non-weight-bearing (NWB), 2D WB) and (2) evaluate the influence of the modality (2D vs. 3D) and the WB condition on the measurements. Three different planning and deformity measurement protocols were analyzed in 19 legs that underwent medial open-wedge high tibial osteotomy (HTO): (1) a 3D WB protocol, after 2D/3D registration of 3D CT models onto the long-leg radiograph (LLR) (3D WB), (2) a 3D NWB protocol based on the 3D surface models obtained in the supine position (3D NWB), and (3) a 2D WB protocol based on the LLR (2D WB). The hip–knee–ankle angle (HKA), joint line convergence angle (JLCA), and the achieved surgical correction were measured for each modality and patient. All the measurement protocols demonstrated excellent intermodal agreement for the achieved surgical correction, with an ICC of 0.90 (95% CI: 0.76–0.96)) (*p* < 0.001). Surgical correction had a higher mean absolute difference compared to the 3D opening angle (OA) when measured with the WB protocols (3D WB: 2.7 ± 1.8°, 3D NWB: 1.9 ± 1.3°, 2D WB: 2.2 ± 1.3°), but it did not show statistical significance. The novel planning modality (3D WB) demonstrated excellent agreement when measuring the surgical correction after HTO compared to existing modalities.

## 1. Introduction

Congenital, degenerative, and posttraumatic deformities of the lower limb are among the most common causes of orthopedic complaints, often affecting young patients. High tibial osteotomy (HTO) is the most frequently performed procedure for deformity correction to restore joint function, relieve joint pain during weight-bearing (WB) activities, and prevent progressive joint degeneration for these conditions [1,2,3,4]. The most common indications for HTO include medial compartment osteoarthritis of the knee accompanied by varus deformity [5], overload of the medial compartment, and spontaneous osteonecrosis of the medial femoral condyle [6]. Additionally, these procedures are frequently performed in association with ligament reconstruction, cartilage repair, and meniscal transplantation in patients with alignment deformity [7,8].

Although satisfactory long-term results after HTO have been reported [9,10], persistent malalignment after surgery is a primary reason for clinical failure [1,11,12,13]. Therefore, accurate preoperative planning and radiographic measurements are mandatory to achieve precise restoration of limb alignment to allow a balanced load distribution across the joint. While realignment surgeries were traditionally planned using two-dimensional (2D) standing long-leg radiographs (LLR), recent technical advances have facilitated computer-assisted planning methods based on patient-specific 3D models, which are generated from non-weight-bearing (NWB) computed-tomography (CT) scans. However, despite such improvements in the field of computer-assisted surgery, the number of outliers after HTO remains unquestionably too high [14]. Potential factors contributing to these outliers include the lack of WB information in 3D planning protocols and the unclear relationship between the bony tibial correction performed and the achieved leg alignment due to additional intra-articular laxity. 

To tackle these problems, Jud et al. investigated the differences in the WB and NWB conditions for the most relevant measurements for preoperative planning in the setting of corrective osteotomies around the knee, such as the hip–knee–ankle angle (HKA) and joint line convergence angle (JLCA) [15], which highlights the importance of WB in the HTO planning process. Additionally, Roth et al. presented a novel 2D/3D registration algorithm, which allows the transfer of patient-specific NWB 3D models into a WB situation., This allows 3D assessment of anatomical deformities and preoperative planning of realignment surgery in WB conditions [16] and embedding the WB state in a fully automated 3D planning algorithm [17]. However, this novel modality (referred to as 3D WB) has neither been validated against existing modalities, such as 3D NWB and conventional 2D planning based on the LLR (2D WB), nor has its accuracy been reported in comparison to the bony opening angle at the tibia. Therefore, the primary aim of this study was to validate a novel planning modality (3D WB) against existing modalities (3D NWB, 2D WB) and to report the differences compared to the opening angle at the tibia for each modality. The secondary aim was to compare the most important pre- and postoperative planning parameters for corrective osteotomies (HKA, JLCA) using 2D and 3D measurement modalities and under WB and NWB conditions. We hypothesized that (1) the measurement protocols demonstrate good intermodal agreement and (2) that the WB condition and modality (3D vs. 2D) significantly influence the planning parameters (HKA, JLCA).

## 2. Materials and Methods

### 2.1. Study Cohort

This study was approved by the institutional review board and the ethical committee (ID: 2017-01616). All the adult patients who underwent medial opening HTO to correct varus alignment for medial degeneration or focal chondral injuries between March 2015 and February 2019, with a complete preoperative and postoperative radiological dataset, were considered as potential candidates for the study. A full radiological dataset comprised a preoperative MRI, pre- and postoperative biplanar standing LLR (representing 2D WB imaging) (EOS imaging system, EOS, Paris, France), as well as a pre- and postoperative CT scans of the lower extremity (Philips Brilliance 64, Philips Healthcare, Best, The Netherlands, or Somatom Definition AS Siemens Healthcare, Erlangen, Germany), representing 3D NWB imaging. Eighteen patients (17 male, 1 female) were included in the study, of which one patient underwent surgery on both legs (19 knees). The mean age was 29 ± 5 years (range: 21–41 years) and the mean body mass index (BMI) was 29.0 ± 4.5 kg/m^2^.

### 2.2. Overview of the Analyzed Leg Alignment Measurement Protocols

**Figure 1:**
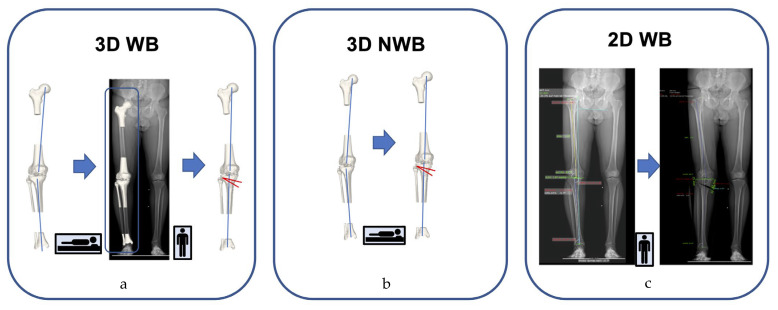
Overview of the three planning modalities investigated. (**a**): Three-dimensional (3D) surface models after 2D/3D registration onto the long-leg radiograph (LLR) to simulate the weight-bearing (WB) state. (**b**): Solely based on 3D non-weight-bearing (NWB) information of 3D surface models obtained in supine position. (**c**): Conventional planning. The red angle demonstrates the opening angle at the tibia.

We analyzed three different planning and deformity measurement protocols, including (1) a 3D WB protocol, after 2D/3D registration of 3D CT models onto the LLR (Figure 1a; referred to as 3D WB), (2) a 3D NWB protocol based on the 3D surface models obtained in a supine position (Figure 1b; referred to as 3D NWB) and (3) a 2D WB protocol based on the LLR (Figure 1c; referred to as 2D WB). 

### 2.3. Validity Testing and Accuracy Assessment

The intermodal agreement of the planning parameters was assessed. All the preoperative and postoperative parameters (HKA, JLCA) were measured for each patient and each modality semi-automatically by blinded observers. The measurement modalities are explained in detail hereafter. The differences between the modalities and the surgical correction compared to the 3D opening angle (3D OA) at the tibia were reported. This allowed us to benchmark the three planning modalities against the “ground truth” of the bony correction at the tibia, as this represents a measurement independent of concomitant intraarticular or ligamentous deformities. The surgical correction of the mechanical axis was defined as follows: Surgical correction = HKA_preoperative_ − HKA_postoperative_. To benchmark the difference compared to the “ground truth”, the mean absolute difference (MAD) between the surgical correction and the 3D OA was calculated for each modality. 

### 2.4. Generation of 3D Triangular Surface Models

In order to create 3D triangular surface models of all the legs (proximal and distal femur, proximal and distal tibia and fibula, as well as the patella and the talus), segmentation of the acquired CT data was conducted using the global thresholding and region growing functionality of standard segmentation software (Mimics Medical 19.0, Materialise NV, Leuven, Belgium). For further analysis, the generated surface models were imported into the planning software CASPA (Computer Assisted Surgery Planning Application version 5.0, Balgrist, University of Zurich, Zurich, Switzerland).

### 2.5. Registration Module for 3D WB Triangular Surface Models

A previously described and validated registration algorithm reported by Roth et al. was used to transfer the generated 3D NWB bone models into the WB state [16]. The registration algorithm was executed using the ImFusion Suite software environment (ImFusion GmbH, Munich, Germany). This so-called intensity-based algorithm uses synthetic 2D images called digitally reconstructed radiographs, which are obtained from NWB CT scans, as well as pre-calibrated biplanar low-dose standing LLR obtained with the EOS imaging system, to generate a transformation matrix, which allows for the above-mentioned transformation of NWB 3D bone models into a WB state. Initially, the registration is initialized through two user-selected annotations. For this, arrows were drawn both on the frontal and lateral EOS radiographs (2D), as well as the CT scan (3D), to connect predefined landmarks on the tibia and the femur. The final registration was then carried out through an optimization routine following a notion of intensity-based 2D/3D registration, resulting in a transformation matrix that describes the relative transformation from the 3D coordinate system of the biplanar EOS to the coordinate system of the CT data [16]. The final transformation of the 3D NWB bone models into the WB state was performed in Matlab (Matlab 2019a, The Math-Works Inc., Natrick, MA, USA) using the above-mentioned transformation matrix.

### 2.6. 3D Measurement (WB and NWB)

**Figure 2:**
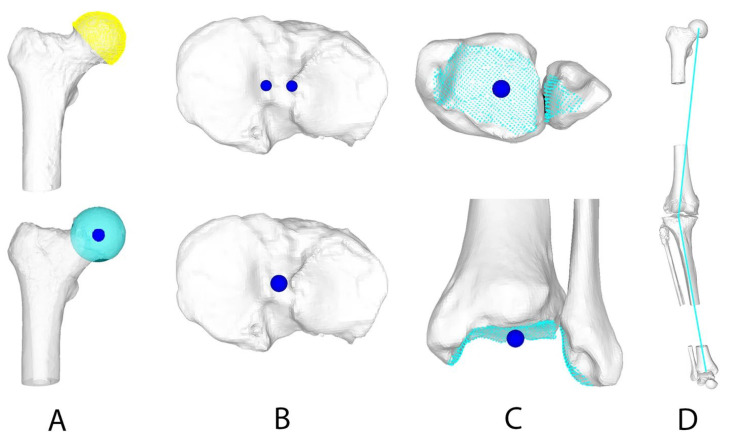
Landmark definition for the mechanical leg axis measurement. (**A**) Definition of the femoral head center (FHC). (**B**) Definition of the knee joint center (KJC). (**C**) Definition of the ankle joint center (AJC). (**D**) Measurement of the hip–knee–ankle angle projected on the frontal plane. Reprinted with permission from Jud et al. [15].

The final 3D deformity assessment of the WB and NWB 3D surface models was carried out using a combination of CASPA and Matlab. After reorientation of the 3D surface models (in CASPA) using a right-handed coordinate system [16], the 3D HKA was measured according to Fürnstahl et al. [18]. Following the definition of the femoral head center (FHC), the knee joint center (KJC), and the ankle joint center (AJC), the HKA was calculated (Matlab) by projecting the angle between a vector connecting the FHC and the KJC and another vector connecting the KJC and the AJC to the frontal plane. This is described in detail by Jud et al. [15] and summarized in Figure 2. 

**Figure 3:**
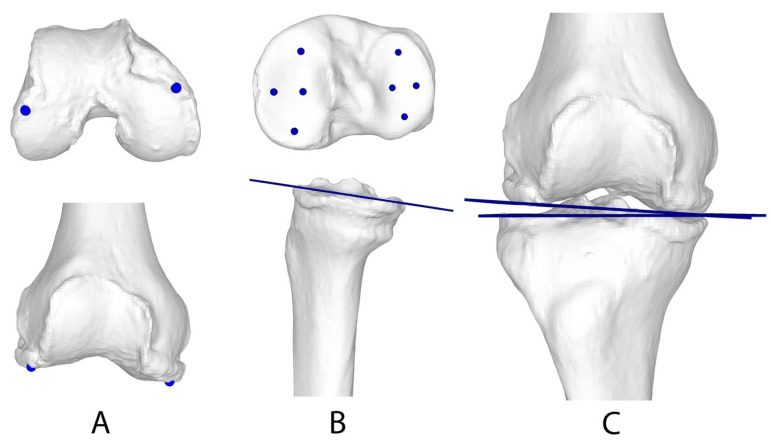
Measurement of the three-dimensional joint line convergence angle. (**A**) Definition of the femoral condyle tangent (FCT). (**B**) The distal tibial condyle tangent (TCT) was defined as the frontal projection of the tibial plateau. (**C**) The 3D JLCA was then calculated as the angle between the FCT and the TCT projected to the frontal plane. Reprinted with permission from Jud et al. [15].

The JLCA was measured as described by Jud et al. [15] using a least squares approach [19], while the tibial plateau plane was defined by ten surface points [15]. The distal tibial condyle tangent (TCT) was then defined as the frontal projection of the tibial plateau. To define the femoral condyle tangent (FCT), the longitudinal axis of the femur was determined using principal component analysis [19]. The 3D JLCA was then calculated as the angle between the FCT and the TCT projected to the frontal plane (Figure 3). 

**Figure 4:**
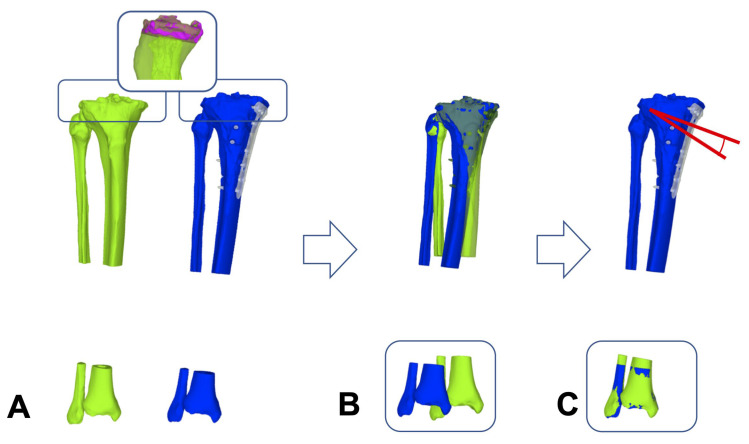
Measurement of the three-dimensional opening angle at the tibia. (**A**) The preoperative 3D surface model (green) and postoperative 3D model (blue) were superimposed proximal at a region defined 15 mm below the joint plane (pink) with the iterative closest point algorithm (ICP). (**B**) Visualizes the proximal superimposition. (**C**) Depicts the subsequent distal superimposition of the preoperative model (green) onto the postoperative model (blue). The 3D opening angle (red) is defined by the rotation of the distal segment in the frontal plane. The red angle demonstrates the opening angle at the tibia.

To measure the 3D OA, the tibia of each 3D bone model was cut 15 mm below the defined joint plane (above the executed osteotomy) to generate individual proximal and distal tibia segments. An iterative closest point (ICP) algorithm was applied to superimpose the proximal tibia segment of the preoperative 3D bone model onto the corresponding proximal tibia segment of the postoperative 3D bone model in such a way that the difference between the two models was as minimal as possible within the selected section [20]. To quantify the bony opening angle at the tibia, the ICP algorithm was re-applied to superimpose the distal tibia segment of the preoperative model onto the postoperative model. The rotation of the distal tibia segment in the frontal plane then defined the 3D OA (Figure 4).

### 2.7. 2D WB Measurements

The conventional planning method was conducted using the LLR in the WB state according to Paley et al. [21] with mediCAD software (version 5.1.0.7, mediCAD Hectec GmbH, Altdorf, Germany). After semi-automatic definition of the FHC, KJC, and AJC, the HKA was calculated automatically by the software. A positive value indicated a varus angle, whereas a negative value indicated a valgus angle. To assess the JLCA, baseline tangents along the femoral and tibial articular surfaces were drawn. The angle measured between these tangents represented the JLCA (positive values indicate lateral opening and negative values indicate medial opening).

### 2.8. Preoperative MRI Evaluation

To analyze the influence of the medial compartment degeneration on the differences between the WB and NWB measurements, the preexisting chondromalacia of the medial compartment was assessed in the coronal plane on fat-saturated proton density MRI sequences modified according to Outerbridge et al. [22]. Furthermore, the medial meniscal extrusion was measured as the horizontal difference between the most medial edge of the meniscus and the tibia on the coronal MRI slice containing the apex of the medial tibial spine (the most medial aspect of the tibia) [23].

### 2.9. Statistical Analysis

Continuous variables are reported as the mean and standard deviation (SD). The normality of the distribution was tested using the Shapiro–Wilk test. Correction of the HKA, from pre- to postoperative, was assessed using Wilcoxon’s test. The intermodal agreement between the measurement modalities (3D WB, 3D NWB, 2D WB) was determined using the intra-class correlation coefficient (ICC), two-way mixed model and absolute agreement and rated according to Fleiss et al. (0.3–0.5: poor; 0.5–0.7 moderate; 0.7–0.9 good; ≥0.9: excellent) [24].

Differences between the 2D vs. 3D modality and WB vs. NWB were analyzed in the pre- and postoperative measurements using a Friedman’s test and post hoc Bonferroni correction. Correlations between the demographics (BMI), radiographic parameters (HKA, meniscus extrusion, Outerbridge score and the MAD of each modality vs. the 3D OA were analyzed using Spearman’s correlation (*r_s_*). *p*-values < 0.05 were considered statistically significant. Statistical analysis was performed using SPSS Statistics (SPSS, IBM Corporation, 1 New Orchard Road Armonk, New York, NY, USA).

## 3. Results

### 3.1. Intermodal Agreement 

**Figure 5:**
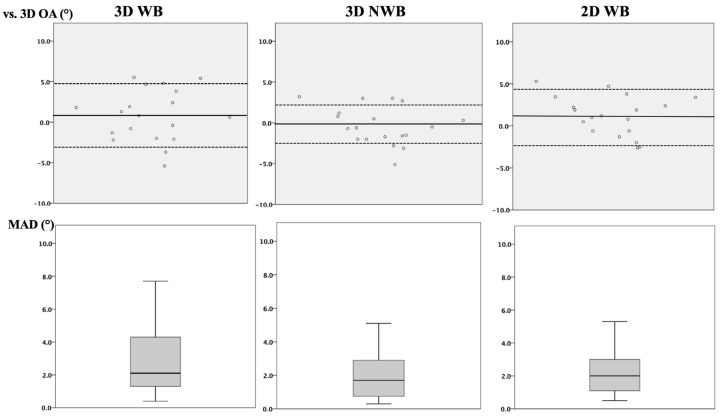
Differences between the surgical correction and the 3D opening angle at the tibia for each modality. **Top row**: The scatterplots depict the difference between the surgical correction and the three-dimensional opening angle at the tibia (3D OA) for each modality (straight line: mean, dotted line: standard deviation). **Bottom row**: Boxplots depict the mean absolute difference (MAD) versus the 3D OA for each modality (n.s.). 3D: Three-dimensional. WB: Weight-bearing. OA: Opening angle. NWB: Non-weight-bearing. 2D: Two-dimensional.

All the measurement protocols demonstrated excellent intermodal agreement for the surgical correction, with an ICC of 0.90 (95% CI: 0.76–0.96)) (*p* < 0.001), and remained good (ICC > 0.70) when each modality was compared to the 3D OA (Table 1). The WB protocols demonstrated a higher MAD between the surgical correction and the 3D OA compared to 3D NWB (n.s.) (Table 1, Figure 5). 

The mean absolute difference between the 3D WB and 3D OA correlated with an increasing preoperative varus deformity (*r_s_*: 0.50; *p* = 0.032) and an increasing BMI (*r_s_*: 0.50; *p* = 0.031). The MAD between the 2D WB and 3D OA correlated with an increasing preoperative varus deformity (*r_s_*: 0.70; *p* = 0.001) and increasing preoperative JLCA (*r_s_*: 0.60; *p* = 0.006). The Outerbridge grade and meniscal extrusion demonstrated no correlation with the reported differences.

### 3.2. Influence of the WB State and Modality (2D vs. 3D) on the Measurement Parameters

The preoperative varus alignment was corrected in all the patients (*p* = 0.001) (Table 1). The pre- and postoperative HKA and the surgical correction tended to be underestimated when assessed using the 3D NWB protocol compared to the WB modalities (Table 1). The JLCA was smaller when assessed in 2D compared to 3D, but it demonstrated no significant differences between the WB and NWB states (Table 2). 

The HKA demonstrated good agreement among the modalities independent of the WB state (all ICC > 0.7) but was significantly smaller in the postoperative 3D NWB (Table 3). The JLCA demonstrated excellent agreement between the 3D WB and 3D NWB, but only moderate agreement compared to the 2D WB (Table 3).

## 4. Discussion

This study aimed to validate a novel planning modality (3D WB) to measure the surgical correction after HTO against existing modalities. The key finding of the study is that the novel planning modality (3D WB) demonstrated excellent agreement compared to existing modalities, with a similar accuracy among all the modalities when benchmarked to the bony OA at the tibia. However, the surgical correction tended to be underestimated in the NWB protocols compared to the WB protocols. This finding emphasizes the importance of accounting for the WB state in deformity assessment and planning of surgical leg realignment procedures.

Three-dimensional surgical planning in limb realignment surgery has become of increasing importance due to the growing automatization of planning protocols and decrease in radiation dose and costs [17,25]. Several studies have investigated the influence of the WB state on deformity measurements and also reported differences between 2D and 3D modalities [15,26]. The integration of the WB state into a 3D planning approach has recently been proposed and validated by Roth et al. [16]. The present study is the first to validate this novel 3D planning protocol by accounting for the WB state in order to measure the surgical correction following limb realignment and to compare it to widely used existing protocols based on 3D NWB imaging or the LLR. In line with previous results, we confirmed the trend that NWB protocols underestimate the deformity and the surgical correction, which emphasizes the importance of including the WB state in 3D planning protocols [15,26]. While 3D planning allows for accurate planning, deformity assessment, and execution using patient-specific guides [27], disregarding the WB status is an apparent drawback. To date, this needs to be considered by the surgeon by assessing the WB information based on 2D imaging as standing X-rays or LLR. This is a cumbersome approach and involves undesired variability. Incorporating the WB status into the 3D planning, however, allows the combination of both techniques’ merits. In this cohort, the proposed workflow to transform the 3D imaging into a WB state has proven to be a valid alternative with similar accuracy as the 3D NWB and 2D WB protocols. This forms the basis for driving automatization and increasing reproducibility and accuracy in the use of 3D leg realignment planning in the future. Incorporating the WB state and 3D anatomy has the potential to improve accuracy and avoid over- and under-correction in HTO in the future.

Furthermore, we were able to benchmark these three planning protocols against the 3D OA. It is known that the relationship between the 3D OA and the achieved surgical correction does not follow a strict 1:1 geometric relationship [28]. This difference can be accentuated due to intraarticular degeneration and joint laxity. It is therefore of the utmost importance to quantify potential factors that distort the targeted correction so that established protocols can be improved. In our cohort, a higher BMI and more extensive varus deformity led to a larger than planned surgical correction (mean absolute difference (MAD) between the surgical correction (HKA_preoperative_ − HKA_postoperative_) and the 3D OA). For the surgeon, this poses a potential risk for over- or under-correction, which can be explained by the increased load shift from medial to lateral with increasing weight. Understandably, this can lead to an undesired opening of the medial joint space. Given that under- or over-correction is associated with impaired clinical outcomes, a refined deformity assessment and improved accuracy of surgical correction planning are highly desired. Especially overweight patients with extensive varus deformities seem to be at risk of malalignment following HTO and might benefit from a planning protocol that includes the WB information. Furthermore, preoperative biomechanical simulation of the planned correction to quantify the joint line opening and load shift should be considered for future projects. Moreover, the deformity cannot be corrected through the true center of the deformity with a simple wedge and the normal alignment of the lower leg cannot always be corrected by performing a single wedge osteotomy, as investigated in this cohort.

When looking at the intermodal agreement of the analyzed measurement parameters, the HKA demonstrated good intermodal agreement among all the modalities. The HKA was underestimated in the NWB protocols relative to the WB protocols, but it reached statistical significance only in the postoperative assessment of the 3D WB vs. 3D NWB. In contrast, the JLCA was not significantly dependent on the WB status but was underestimated in the 2D modalities compared with 3D measurement. This is consistent with previous findings, and the surgeon must be aware of these intermodal differences [15].

This study should be interpreted in the light of its potential limitations. First, only a small number of patients could be included in the study due to the limited availability of pre- and postoperative biplanar standing long-leg EOS radiographs, as this imaging modality is not yet used by default in everyday clinical practice. Second, the accuracy of the algorithm used to create the WB 3D models is limited by the quality of the biplanar EOS scans, including the balanced load distribution and leg position, as reported in a previous study [16]. Nevertheless, Roth et al. demonstrated excellent reliability for the use of the registration algorithm [16]. Third, the clinical benefits of including the WB state in current 3D protocols cannot be derived from the current study and warrant further research.

## 5. Conclusions

The novel planning modality (3D WB) demonstrated excellent agreement when measuring the surgical correction after HTO compared to existing modalities. However, the leg alignment and surgical correction tended to be underestimated in the NWB compared to WB protocols. An increasing difference between the surgical correction and 3D OA was associated with more extensive varus deformities and a higher BMI, representing a risk of over- or under-correction in this patient cohort. 

## Figures and Tables

**Table 1:** Intermodal agreement and mean absolute difference compared to the 3D opening angle.

	3D-WB vs. 3D OA	3D-NWB vs. 3D OA	2D-WB vs. 3D OA	*p*-Value
**ICC** (95% CI)	0.71 (0.29–0.89) ^+^	0.84 (0.60– 0.94) *	0.74 (0.32–0.90) ^x^	+ = 0.005; * < 0.001; x = 0.002
**MAD** (°)	2.7 ± 1.8	1.9 ± 1.3	2.2 ± 1.3	(n.s.)

ICC: Intraclass correlation coefficient. CI: Confidence interval. MAD: Mean absolute difference. 3D Three-dimensional. WB: Weight-bearing. OA: Opening angle. NWB: Non-weight-bearing. 2D: Two-dimensional. Significant *p*-values (<0.05) are marked in bold. +, *, x indicate respective *p*-value.

**Table 2:** Deformity assessment and achieved surgical correction among all the modalities.

	3D-WB	3D NWB	2D WB	3D Opening Angle	*p*-Value
HKA (°)					
Preoperative	6.6 ± 4.4	6.0 ± 2.6	7.0 ± 3.3	-	(n.s.)
Postoperative	−2.1 ± 2.3 *	−1.2 ± 1.9 *	−2.0 ± 1.6	-	* 0.014
Achieved surgical correction	8.7 ± 4.9	7.2 ± 3.2	9.1 ± 3.6	7.6 ± 2.9	(n.s.)
JLCA (°)					
Preoperative	3.9 ± 2.2 *	3.3 ± 2.1	2.6 ± 2.3 *	-	***p* = 0.011**
Postoperative	3.4 ± 2.0	3.5 ± 2.0	1.8 ± 1.3	-	(n.s.)

HKA: Hip–knee–ankle angle. WB: Weight-bearing. NWB: Non-weight-bearing. JLCA: Joint line convergence angle. *p*-value: Friedman’s test (post hoc Bonferroni corrected). Significant *p*-values (<0.05) are marked in **bold**. * marks significant group comparisons.

**Table 3:** Overview of the intermodality agreement for the pre- and postoperative mechanical axis and joint line convergence angle.

	3D WB vs. 3D NWB	3D WB vs. 2D-WB	3D NWB vs. 2D WB
**HKA preoperative**			
ICC (95% CI)	0.88 (0.70–0.95) **(*p* < 0.001)**	0.91 (0.79–0.97) **(*p* < 0.001)**	0.89 (0.64–0.96) **(*p* < 0.001)**
MAD: Mean ± SD	1.9 ± 1.5	1.5 ± 1.6	1.5 ± 1.2
*p*-value (Friedman’s)	*p* = 0.12	*p* = 0.12	*p* = 0.12
**JLCA preoperative**			
ICC (95% CI)	0.94 (0.70–0.98) **(*p* < 0.001)**	0.62 (0.06–0.85) **(*p* = 0.01)**	0.57 (−0.08–0.83) **(*p* = 0.039)**
MAD: Mean ± SD	0.9 ± 0.6	2.1 ± 2.5	2.0 ± 1.5
*p*-value (Friedman’s)	*p* = 0.155	***p* = 0.011**	*p* = 0.991
**HKA postoperative**			
ICC (95% CI)	0.78 (0.40–0.91) **(*p* < 0.001)**	0.84 (0.57–0.94) **(*p* < 0.001)**	0.87 (0.43–0.96) **(*p* < 0.001)**
MAD: Mean ± SD	1.5 ± 1.2	1.1 ± 1.0	0.9 ± 0.8
*p*-value (Friedman’s)	***p* = 0.014**	*p* = 1.00	*p* = 0.069
**JLCA postoperative**			
ICC (95% CI)	0.96 (0.96–0.99) **(*p* < 0.001)**	0.64 (0.12–0.86) **(*p* = 0.013)**	0.53 (−0.13–0.82) **(*p* = 0.048)**
MAD: Mean ± SD	0.4 ± 0.3	2.0 ± 1.3	2.0 ± 1.4
*p*-value (Friedman’s)	*p* = 0.241	*p* = 0.241	*p* = 0.241

ICC: Intra-class correlation coefficient. 2D: Two-dimensional. 3D: Three-dimensional. WB: Weight-bearing. NWB: Non-weight-baring. HKA: Hip–knee–ankle angle; JLCA: Joint line convergence angle. MAD: Mean absolute difference. SD: Standard deviation. CI: Confidence interval. Significant *p*-values (<0.05) are marked in **bold**.

## Data Availability

Data are available upon reasonable request.
